# Association of serum testosterone concentrations with central adiposity in men who inject heroin in Mombasa County, Kenya

**DOI:** 10.11604/pamj.2026.53.164.49885

**Published:** 2026-04-19

**Authors:** Josephine Wambani, Abel Odhiambo Onyango, Christine Wanjala, Valentine Budambula, Aabid Abdulmajid Ahmed, Gerald Juma, Tom Were

**Affiliations:** 1 Department of Medical Laboratory Sciences, Masinde Muliro University of Science and Technology, Kakamega, Kenya,; 2 Department of Medical Laboratory Sciences, Kabarak University, Nakuru, Kenya,; 3 Department of Biochemistry, Microbiology and Biotechnology, Kenyatta University, Nairobi, Kenya,; 4 Department of Public and Community Health, Technical University of Mombasa, Mombasa, Kenya,; 5 Department of Clinical Research Administration, Bomu Hospital, Mombasa, Kenya,; 6 Department of Biochemistry, University of Nairobi, Nairobi, Kenya,; 7 Department of Human Pathology, Masinde Muliro University of Science and Technology, Kakamega, Kenya

**Keywords:** Heroin injection, serum testosterone, occupation, central adiposity

## Abstract

**Introduction:**

heroin is the most commonly injected drug among people who inject drugs globally. Heroin injection disrupts the hypothalamic-pituitary axis, leading to hormonal imbalances such as altered testosterone concentrations in men. Men who inject heroin concurrently use other substances, which may further affect testosterone levels. Lower testosterone levels are associated with increased central adiposity and decreased sexual function. This study aimed to determine how substance use patterns and adiposity markers are associated with testosterone concentrations among heroin-injecting men in Mombasa County.

**Methods:**

in this analytical cross-sectional study, serum samples from male participants with a documented history of substance use were included. Exclusion criteria included HIV-positive status and incomplete data. Demographic, substance use profiles, and clinical data were extracted from existing records. Serum total testosterone levels were measured using a chemiluminescence immunoassay technique. Descriptive statistics, non-parametric between-group comparisons, Spearman correlations, and multivariable linear regression were used to analyze the data, with statistical significance set at p < 0.05.

**Results:**

relatively higher median testosterone concentrations were observed in heroin-injecting men (3.570 nmol/L) compared to non-heroin-injecting men (0.097 nmol/L) (p < 0.0001). Hypogonadism, defined as testosterone below 8.0 nmol/L, was present in 97.9% of heroin-injecting men and in all non-heroin-injecting men (100.0%), with no significant difference between the two groups (p = 0.712). Heroin-injecting men working in small businesses and transport sectors had relatively higher testosterone levels compared to those working in the hospitality sector (p = 0.001). Hip circumference was positively correlated with testosterone levels in the heroin-injecting men, with a significant positive correlation (Spearman's ρ = 0.173, p = 0.039). Similarly, waist-to-hip ratio showed a significant negative correlation in the heroin-injecting men (Spearman's ρ = -0.204, p = 0.014). On multivariable linear regression, employment in the small business sector (B = 3.147, p < 0.001) and transport sector (B = 3.103, p < 0.001), duration of heroin injection of one year or more (B= 0.802, p = 0.012), and waist-to-hip ratio (B= -6.146, p = 0.006) were independent predictors of serum total testosterone concentrations (Adjusted R^2^= 0.201).

**Conclusion:**

this study reports that serum total testosterone concentrations were significantly higher in heroin-injecting men than in non-heroin-injecting men, though both groups fell below the clinical threshold for hypogonadism. Multivariable regression analysis identified employment in the small business and transport sectors, prolonged heroin injection of at least one year, and waist-to-hip ratio as independent predictors of serum total testosterone concentrations. Cigarette smoking was independently associated with lower testosterone after adjustment, though this association warrants cautious interpretation. These findings emphasize the need for integrated interventions that address both the endocrine consequences of heroin injection and the occupational and lifestyle factors that modulate testosterone in this population.

## Introduction

Heroin use constitutes a major public health challenge worldwide, with significant health, social, and economic consequences [1,2]. Among people who inject drugs (PWID) globally, an estimated 78.1% have used heroin, with 71.8% reporting having used it through injection [3]. In sub-Saharan Africa, heroin use is a growing concern. For instance, in Tanzania, heroin accounted for 12.6% of the total incidents in a study investigating the changes in illegal substances availability on the Tanzanian mainland [4]. In South Africa, heroin use is prevalent, with a significant portion of users attending rehabilitation centers [5]. In Kenya, heroin use is most prevalent among PWID, with injection reported at particularly high rates in Nairobi and along the Coast region [6]. In Mombasa, heroin use has remained a major public health concern for more than 25 years, marked by a significant shift in consumption patterns from inhalation to injection beginning in the late 1990s [7]. In this region, heroin is one of the most commonly abused substances, affecting individuals across various socio-economic backgrounds [8]. The rise in heroin consumption is linked to Mombasa's role as a transit point for drug trafficking, which has contributed to the increasing availability and use of heroin in the area [9]. Heroin use has been associated with disruptions in endocrine function, particularly circulating testosterone levels [10], but findings are inconsistent.

Testosterone plays a vital role in maintaining muscle mass, bone density, mood regulation, energy levels, and reproductive health [11]. Studies have indicated that heroin use significantly lowers testosterone levels in the blood, with recovery occurring after prolonged abstinence [10]. However, the degree of suppression and the recovery timeline can vary. For instance, some studies have indicated that testosterone levels can remain suppressed during methadone maintenance therapy [12]. In the context of androgen abuse rather than opioid use, testosterone levels have been reported to recover within approximately three months of abstinence [13]. Additionally, the relationship between heroin dosage and testosterone suppression is not always consistent, with some studies showing a dose-related pattern [14]. Studies on heroin's effects on testosterone levels have yielded mixed results, with some studies showing suppression of testosterone [10]. Other studies indicate elevated levels in opioid users [15] and among heroin users, particularly those concurrently smoking tobacco [16]. Poly-drug users are likely to experience changes in testosterone levels. For example, chronic alcohol consumption has been associated with lower testosterone levels in adult men [17]. Similarly, cannabis use has been linked to decreased testosterone, primarily through mechanisms involving oxidative stress and testicular dysfunction [18]. However, the trend is not universal. For example, a cohort study conducted at a single academic andrology centre observed higher mean serum testosterone among cannabis users [19].

In addition, the influence of tobacco use on testosterone levels remains inconsistent. One cross-sectional, population-based study reported lower total serum testosterone in smokers [20], while another investigation in both men and women aged 18-62 years found elevated testosterone levels among tobacco users [16]. Beyond substance use, central adiposity is another modulator of testosterone concentrations in men. Studies demonstrate that testosterone levels are associated with fat distribution, particularly central adiposity [21,22]. A number of markers are used to evaluate central adiposity, including waist-to-hip ratio (WHR) [23], hip circumference (HC), waist circumference (WC) [24], and body mass index [25]. Although body mass index (BMI) is a general measure of obesity [26], it can also infer central adiposity [27]. Lower testosterone levels are associated with higher BMI and central fat deposition in men [28,29]. Similarly, lower testosterone levels are associated with higher waist-to-hip ratio [30] and waist circumference [31], indicating increased central adiposity. Previous studies have shown that small businesses often serve as fronts for drug-dealing activities. Research in Mexico City revealed that low-level drug dealers incorporated illicit product sales into licit companies [32]. The study found that the “front businesses” provided a cover for the selling of illegal drugs [32]. In Uganda, alcohol was sold 24/7 in markets and streets, according to research. Alcohol availability increased due to sophisticated marketing efforts that targeted young people in ways unacceptable in higher-income countries. To sell their products, multinational marketing and advertising firms sponsored popular events like football matches [33].

Substance abuse among individuals in the transport sector is common and is influenced by various factors, primarily related to coping mechanisms and job demands [34,35]. Most commercial bus drivers and road transport workers use drugs to manage stress and anxiety [36,37]. Another motive for substance usage is to stay alert while driving and improve work performance [37]. Transport workers may also use psychoactive drugs to manage depression and daily life challenges. This can cause mood swings and substance abuse. After a long day, substance usage is driven by the urge to unwind [36]. Heroin injection is linked to hormonal imbalances [38] including low serum testosterone levels [10] and central adiposity [39]. Most of these findings originate from high-income countries, with little data from sub-Saharan Africa. To our knowledge, no research has examined testosterone levels or central adiposity markers in heroin-injecting men in Kenya, particularly in Mombasa County where heroin use is high [40]. Therefore, this study aimed to determine how substance use patterns and adiposity markers are associated with serum total testosterone concentrations among heroin-injecting men in Mombasa County. Specifically, the study was guided by the following research questions: i) What are the serum total testosterone concentrations among heroin-injecting men compared to non-heroin-injecting men in Mombasa County, Kenya; ii) Among heroin-injecting men, which factors are independently associated with serum total testosterone concentrations after adjusting for age, BMI, cigarette smoking, and occupational category; iii) What is the central adiposity status of heroin-injecting men in Mombasa County, and how do central adiposity markers correlate with serum total testosterone concentrations; iv) What are the substance use profiles of heroin-injecting men, and how do these profiles relate to testosterone concentrations in this population?

## Methods

**Study design:** this was an analytical cross-sectional study.

**Settings:** this study was conducted at Bomu Hospital, a social enterprise facility in Mombasa, Kenya. Conducting the study at Bomu Hospital in Mombasa was advantageous, given the hospital´s established role in supporting research involving PWID, as demonstrated by its involvement in PWID nutritional studies in Coastal Kenya [41].

**Study timeline:** the parent study was conducted between July 2012 and February 2013. Archived records and serum samples were accessed in September 2025 following receipt of ethical and regulatory approvals. Laboratory analysis of serum testosterone was completed in September 2025 at Kenyatta National Hospital.

**Study size:** the desired level of accuracy and the nature of the study informed the sample size for the study. The sample size was determined using the following formula [42].


$$n=\frac{Z^{2}\times p\times q\times N}{e^{2}\left(N-1\right)+Z^{2}\times p\times q}$$

Where Z = 1.96, representing the standard variate at the 95% confidence level; p = 0.12, representing the estimated prevalence of people who inject drugs (PWID) in Kenya as reported by a global consortium study [43]; q = 0.88, representing (1 - p); e = 0.05, representing the acceptable margin of error at 5% precision; and N = 30,502, representing the estimated total population of PWID in Kenya at a prevalence of 12% [43]. Substituting these values into the formula yielded a minimum required sample size of 160 participants. The 160 participants were subsequently stratified proportionately into heroin-injecting and non-heroin-injecting groups, based on the documented prevalence of heroin injection among PWID drawn from published regional studies. Specifically, a rapid situational assessment conducted among PWID in Nairobi and coastal Kenya, the two regions with the highest burden of injection drug use in the country, reported that heroin was the most commonly injected substance, with a prevalence of 98% [44]. A separate study conducted among PWID in Addis Ababa, Ethiopia, reported a heroin injection prevalence of 79% [45]. The average of the two regional prevalence estimates was used as an approximate pooled proportion to guide proportional stratification as follows: (98% + 79%)/2= 88.5%, rounded to 89%. The average prevalence of 89% was applied to the total sample of 160 to determine the number of heroin-injecting participants: 89% x160 = 142.4, rounded to 143. The remaining participants constituted the non-heroin-injecting group: 160-143 = 17. The Kenyan estimate alone was not used as it would have yielded only three non-injecting participants, an insufficient number for any between-group comparison; accordingly, the Ethiopian estimate was incorporated to produce a minimum viable reference group, with the understanding that the primary analytical population remains the heroin-injecting group of 143 men.

**Participants:** the present study used archived serum samples from a larger parent investigation that enrolled 752 adults across multiple drug-use categories at Bomu Hospital and its branches in Kisauni, Likoni and Jomvu sub-counties of Mombasa County between July 2012 and February 2013, from which multiple analytical subsets have been published [8,41,46]. Injection drug users in the parent study were recruited using three complementary non-probability sampling strategies. Respondent-driven sampling was initiated with three seeds, each a known drug user receiving addiction counselling at Bomu Hospital. Each seed received three uniquely coloured coupons to recruit peers within Mombasa County, constituting the first recruitment wave. Participants who completed interviews in each wave received six coupons for further peer recruitment, with successive waves continuing until recruitment stalled (no more participants were being enrolled). Snowball sampling and makeshift outreach were then employed, whereby a rehabilitated former injection drug user directly recruited additional participants from known drug congregation points within the county [8]. The parent cohort included 371 injection drug users, of whom 213 were HIV-negative [41]. From this HIV-negative injection drug user pool, archived serum samples from male participants with a documented history of substance use, visible needle scars and complete demographic and clinical data were eligible for inclusion in the present study. Participants who were HIV-positive, female, or had incomplete data were excluded. HIV-positive individuals were specifically excluded because HIV infection disrupts the hypothalamic-pituitary-gonadal axis through multiple mechanisms, and antiretroviral therapy introduces further hormonal variability [47]. A total of 160 participants met all inclusion criteria and were enrolled. The defining criterion for heroin-injecting men was active heroin injection, while the defining criterion for non-heroin-injecting men was the absence of heroin injection, despite possible use of other substances. Both groups were drawn from the same pool of drug-using men in Mombasa County.

**Variables:** demographic data, including age (years), height (meters), weight (kilograms), body mass index (BMI), mid-upper arm circumference (MUAC), waist circumference (WC), hip circumference (HC), chest circumference (CC), chest-to-waist ratio (CWR), and waist-to-hip ratio (WHR) were extracted from archived records. Additionally, information regarding occupation, education level, marital status, and sexual orientation was also retrieved. Data on substance use profiles, including heroin, alcohol, cannabis, brown sugar, cigarettes, cocaine, cocktail, and khat, and the duration and frequency of drug injection were also extracted and documented. Socio-demographic and clinical information were originally collected using a participant-assisted questionnaire, administered by trained personnel [8].

**Measurement of testosterone concentrations:** archived serum samples from the parent study (collected between July 2012 and February 2013) were stored at -80°C at Bomu Hospital until retrieval. The serum samples were stored at -80°C in single-use aliquots prior to analysis. This approach preserves testosterone integrity over prolonged storage, consistent with established biobanking standards [48]. Samples were retrieved in September 2025 following institutional ethical approval (MMUST/ISERC/095/2025) and NACOSTI research clearance. Testosterone assays were performed in September 2025 at Kenyatta National Hospital. Serum total testosterone levels were determined using the Roche Cobas e601 Analyzer, which employs a competitive electrochemiluminescence immunoassay technique. Serum samples were thawed under controlled laboratory conditions and processed immediately. Storage in single-use aliquots precluded repeated freeze-thaw cycles and preserved sample integrity. In the assay, a 20µL aliquot of serum was first incubated with a biotinylated monoclonal antibody specific to testosterone. Next, a ruthenium-labeled testosterone analogue and streptavidin-coated microparticles were introduced, allowing immune complexes to form and bind to a solid phase via the high-affinity biotin-streptavidin interaction. These complexes were then magnetically captured on an electrode surface, washed to remove unbound components, and subjected to a voltage that induced a chemiluminescent signal. The emitted light, inversely proportional to the testosterone concentration, was detected by a photomultiplier and quantified against a calibration curve derived from manufacturer-supplied standards and the instrument’s internal master curve. Testosterone concentrations, reported in nanomoles per liter (nmol/L), were reviewed for consistency. Detailed records were maintained for traceability and reproducibility.

**Bias:** to minimize selection bias, both groups were drawn from the same drug-using population. Laboratory personnel were blinded to participants’ heroin injection status to reduce measurement bias.

**Quantitative variables:** continuous variables, including age, body mass index (BMI), mid-upper arm circumference (MUAC), waist circumference (WC), hip circumference (HC), chest circumference (CC), chest-to-waist ratio (CWR), waist-to-hip ratio (WHR), and serum total testosterone levels, were analysed as continuous data and summarized using medians and interquartile ranges (IQRs). Although BMI is a general measure of adiposity rather than a specific marker of central fat distribution, it was included as a covariate in the regression model alongside WHR. Body mass index status was categorised as underweight (< 18.5 kg/m^2^) or normal weight (≥ 18.5 kg/m^2^), consistent with established BMI classification criteria [49]. Hypogonadism was defined by testosterone levels of <8.0 nmol/L [50]. These thresholds were derived from established reference ranges in regional clinical studies and enabled categorical analysis of testosterone status. Categorical variables (occupation, education level, marital status, sexual orientation, and substance use categories) were presented as numbers (n) and proportions (%).

**Statistical methods:** data (demographic measures, socio-demographic characteristics, substance use profiles, and total testosterone levels) were entered, cleaned, coded, and analyzed using the Statistical Package for the Social Sciences (SPSS), version 23 (IBM, Chicago). All the tested archived serum samples had complete demographic and clinical data. Participants were recruited using non-probability sampling methods, including snowball sampling, respondent-driven sampling, and outreach methods. We acknowledge the potential for selection bias and the limited generalizability of our findings. Descriptive statistics were performed to summarize demographic, clinical, and substance use profiles. Continuous variables (age, BMI, MUAC, WC, HC, CC, CWR, WHR, and total testosterone levels) were reported as medians and interquartile ranges (IQRs), while categorical variables (occupation, education level, marital status, sexual orientation, and substance use categories) were presented as numbers (n) and proportions (%). Group comparisons for continuous variables were performed using the Mann-Whitney U test, while categorical variables were compared using Fisher's exact test or the chi-square test. Statistical significance was set at p < 0.05. To evaluate differences in testosterone levels across groups, Fisher's exact test (for hypogonadism) and chi-square tests were performed, with significance defined by p < 0.05. Spearman’s correlation coefficients (Spearman’s ρ) were used to assess associations between continuous variables and testosterone levels, with corresponding p-values.

The Kruskal-Wallis test was performed to examine the relationship between occupation and testosterone levels across groups. Post hoc pairwise comparisons were conducted using Dunn’s test with a Bonferroni correction for multiple comparisons, with significance maintained at p < 0.05. For the duration of heroin injection, statistical significance was assessed using the Mann-Whitney U test, with a significance threshold of p < 0.05. No sensitivity analyses were performed. All variables analyzed were complete with no missing data, and inspection of the data did not identify extreme outliers that would warrant alternative model specifications. Non-parametric tests were selected throughout because they do not assume equal group sizes or normally distributed data. To identify independent predictors of serum total testosterone concentrations among heroin-injecting men, a multivariable linear regression analysis was performed using the forced entry method (Enter). The following variables were entered simultaneously as covariates: age (years), body mass index (kg/m^2^), cigarette smoking (current smoker coded as 1 versus non-smoker coded as 0), occupational category (small business sector versus hospitality sector as reference category; transport sector versus hospitality sector as reference category), duration of heroin injection (one year or more coded as 1 versus less than one year coded as 0), and waist-to-hip ratio. Dummy coding was applied to occupational category, with the hospitality sector serving as the reference group. Absence of multicollinearity was assessed using the variance inflation factor (VIF), with a threshold of 5.0 used to identify unacceptable collinearity. Model fit was assessed using the adjusted coefficient of determination (Adjusted R^2^) and the overall F test.

**Ethical considerations:** this study adhered to the principles outlined in the Declaration of Helsinki. Ethical approval for the study was obtained from the Masinde Muliro University of Science and Technology Institutional Ethics Review Committee (MMUST/ISERC/095/2025). As secondary data were utilized, direct consent from individual participants was not required; however, the original study was approved and had obtained broad written informed consent from participants prior to data collection. Informed consent procedures had included participant education on study aims, voluntary participation, the right to withdraw, and the potential risks of substance use. Throughout the study, participant confidentiality was strictly maintained, and data were handled to ensure anonymity, except where information was needed for clinical care purposes in the primary study. Participants benefited from the original study through access to health education, counselling, and referrals for clinical care when health issues or substance use risks were identified.

## Results

**Participants:** a total of 160 HIV-negative male participants met all inclusion criteria and were enrolled, with no exclusions at any stage. Of these, 143 (89%) were heroin-injecting men and 17 (11%) were non-heroin-injecting men.

**Sociodemographic and substance use profiles of study participants:** all variables including demographic (age, education level, marital status, occupation, sexual orientation), clinical (height, weight, BMI, MUAC, waist circumference, hip circumference, chest circumference, chest-to-waist ratio, waist-to-hip ratio), and substance use profiles (heroin, alcohol, cannabis, brown sugar, cigarettes, cocaine, cocktail, khat) were complete for all the study participants (n = 160). No missing data were encountered for any variable. The sociodemographic characteristics and substance use profiles of heroin-injecting men (n=143) compared to non-heroin-injecting men (n=17) are outlined in Table 1. Analysis of education levels among the study participants showed no significant differences between heroin and non-heroin-injecting men in both basic education (65.0% vs. 76.5%) and higher education (35.0% vs. 23.5%) (p = 0.425). Although a majority of the participants were single (76.9% vs. 64.7%), the difference in the proportion of married individuals (23.1% vs. 35.3%) between heroin and non-heroin-injecting men was similar (p = 0.368). A significant difference was found in occupation categories. Most of the heroin-injecting men were involved in small businesses (49.0% vs. 35.3%) and the transport sector (44.8% vs. 35.3%), while non-heroin-injecting men were working in hospitality (29.4% vs. 6.3%) (p = 0.006). There were significant differences in sexual orientation between the two groups. A higher percentage of heroin-injecting men identified as heterosexual (76.9% vs. 47.1%), while a larger proportion of non-heroin-injecting men identified as homosexual (14.0% vs. 23.5%) and bisexual (9.1% vs. 29.4%) (p = 0.015). Duration of injection differed significantly between groups, with 68.5% of heroin-injecting men and 94.1% of non-heroin-injecting men reporting injection histories of ≥1 year (p = 0.026). Regarding substance use, significant differences were observed. Cigarette use was significantly higher among heroin-injecting men (70.6% vs. 41.2%) (p = 0.025). There was no significant difference in cannabis use between the groups, with 52.4% of heroin-injecting men and 41.2% of non-heroin-injecting men reporting use (p = 0.447). Although not statistically significant, a smaller proportion of heroin-injecting men reported using a cocktail (24.5% vs. 47.1%) (p = 0.078).

**Table 1:** sociodemographic characteristics and substance use profiles of heroin-injecting (n=143) and non-heroin-injecting (n=17) men in Mombasa County, Kenya, July 2012 to February 2013

Variable	Non-heroin-injecting men, n=17	Heroin-injecting men, n=143	P-value
**Education**			
Basic	13 (76.5)	93 (65.0)	0.425
Higher	4 (23.5)	50 (35.0)	
**Marital status**			
Single	11 (64.7)	110 (76.9)	0.368
Married	6 (35.3)	33 (23.1)	
**Occupation**			
Hospitality	5 (29.4)	9 (6.3)	**0.006**
Small business	6 (35.3)	70 (49.0)	
Transport	6 (35.3)	64 (44.8)	
**Sexual orientation**			
Homosexual	4 (23.5)	20 (14.0)	**0.015**
Bisexual	5 (29.4)	13 (9.1)	
Heterosexual	8 (47.1)	110 (76.9)	
**Duration of injection, years**			
<1 year	1 (5.9)	45 (31.5)	**0.026**
≥1 year	16 (94.1)	98 (68.5)	
Cigarette	7 (41.2)	101 (70.6)	**0.025**
Cannabis	7 (41.2)	75 (52.4)	0.447
Cocktail	8 (47.1)	35 (24.5)	0.078
Alcohol	1 (5.9)	31 (21.7)	0.198
Brown sugar	2 (11.8)	22 (15.4)	0.999
Khat	9 (52.9)	22 (15.4)	**0.001**
Cocaine	13 (76.5)	0 (0.00)	**0.001**

Categorical variables are expressed as numbers (n) and proportions (%); for categorical variables, Fisher's exact test was used for 2x2 tables, and the chi-square test was conducted for occupation and sexual orientation. Significant differences are marked by P-values less than 0.05. Values in bold are significant P-values

The proportion of alcohol use was higher among heroin-injecting men (21.7% vs. 5.9%), but this difference was not statistically significant (p = 0.198). 15.4% of heroin-injecting men reported using brown sugar, while 11.8% of non-heroin-injecting men reported the same, showing no significant difference (p = 0.999). Khat use was significantly higher among non-heroin-injecting men (52.9% vs.15.4%) (p = 0.001). Non-heroin-injecting men exclusively reported cocaine use (76.5% vs. 0%), with a significant difference (p = 0.001). Table 2 presents anthropometric and physical characteristics of the study participants. Heroin-injecting men had a slightly higher median age (33.0 years) compared to non-heroin-injecting men (32.5 years), although this difference was not statistically significant (p = 0.310). Similarly, there was no significant difference in height between the two groups (1.71 m vs. 1.74 m, p = 0.201). In terms of weight, heroin-injecting men had a lower median weight (54.0 kg vs 59.0 kg), but the difference was not statistically significant (p = 0.128). Both groups had an identical median BMI of 18.69 kg/m^2^, with no significant differences in BMI status between the groups (p = 0.999). For BMI status, 45.5% of heroin-injecting men were underweight (BMI <18.5 kg/m^2^) vs. 41.2% of non-heroin-injecting men, with no significant difference (p = 0.801). Median body measurements were similar between the two groups. For CC, the median was 85.0 cm (IQR 7.0) in the heroin-injecting group versus 86.0 cm (IQR 10.5) in the comparison group (p= 0.233). Mid-upper arm circumference was 26.0 cm (IQR 3.0) versus 27.0 cm (IQR 3.0) (p = 0.803). Waist circumference was 75.0 cm (IQR 6.0) versus 76.0 cm (IQR 4.5) (p = 0.621). Hip circumference was 91.0 cm (IQR 7.0) versus 91.5 cm (IQR 7.5) (p = 0.382). Chest-to-waist ratio medians were 1.14 (IQR 0.09) versus 1.13 (IQR 0.08) (p = 0.807), and WHR were 0.83 (IQR 0.06) versus 0.82 (IQR 0.08) (p = 0.665).

**Table 2:** anthropometric and physical characteristics of heroin-injecting (n=143) and non-heroin-injecting (n=17) men in Mombasa County, Kenya, July 2012 to February 2013

Variable	Non-heroin-injecting men, n=17	Heroin-injecting men, n=143	P-value
Age, years	32.5 (8.7)	33.0 (10.3)	0.310
Height, m	1.74 (0.082)	1.71 (0.087)	0.201
Weight, kg	59.0 (10.0)	54.0 (8.0)	0.128
BMI, kg/m^2^	18.69 (2.99)	18.69 (2.42)	0.999
**BMI status**			
<18.5	7 (41.2)	65 (45.5)	0.801
≥18.5	10 (58.8)	78 (54.5)	
MUAC, cm	27.0 (3.00)	26.0 (3.00)	0.803
Chest circumference, cm	86.0 (10.5)	85.0 (7.00)	0.233
Waist circumference, cm	76.0 (4.50)	75.0 (6.00)	0.621
Hip circumference, cm	91.5 (7.50)	91.0 (7.00)	0.382
CWR	1.13 (0.08)	1.14 (0.09)	0.807
WHR	0.82 (0.08)	0.83 (0.06)	0.665

Results are presented as the median with interquartile range (IQR) for continuous variables and as numbers (n) and proportions (%) of individuals for categorical variables, BMI: body mass index, MUAC: mid-upper arm circumference, CWR: chest-to-waist ratio, WHR: waist-to-hip ratio. BMI status: underweight, BMI < 18.5 kg/m^2^; and normal weight, BMI ≥ 18.5 kg/m^2^; Mann-Whitney U test was used for comparing differences in continuous variables, while the Fisher’s exact test was used to compare the distribution of the BMI status between heroin and non-heroin-injecting men

**Serum testosterone concentrations and hypogonadism status:** Table 3 presents the distribution of serum testosterone levels and hypogonadism status among the study participants. Higher median testosterone concentrations were observed in heroin-injecting men (3.570 nmol/L, IQR 1.690) compared to non-heroin-injecting men (0.097 nmol/L, IQR 2.752), and this difference was statistically significant (p < 0.0001). Hypogonadism, defined as testosterone below 8.0 nmol/L [50], was present in 140 of 143 heroin-injecting men (97.9%) and in all 17 non-heroin-injecting men (100.0%). The difference in hypogonadism prevalence between the two groups was not statistically significant (p = 0.712). Only three heroin-injecting men (2.1%) had testosterone concentrations at or above the hypogonadism threshold.

**Table 3:** distribution of serum testosterone levels and hypogonadism status among heroin-injecting (n=143) and non-heroin-injecting (n=17) men in Mombasa County, Kenya, July 2012 to February 2013

Variable	Non-heroin-injecting men, n=17	Heroin-injecting men, n=143	P
Testosterone, nmol/L	0.097 (2.752)	3.570 (1.690)	**< 0.0001**
**Hypogonadism status**			
Hypogonadal (<8.0 nmol/L)	17 (100.0)	140 (97.9)	0.712
Not hypogonadal (≥8.0 nmol/L)	0 (0.0)	3 (2.1)	

Results are presented as median with interquartile range (IQR) for continuous variables and as numbers (n) and proportions (%) for categorical variables. For continuous variables, P-values were calculated using the Mann-Whitney U test. For categorical variable (hypogonadism), p-values were calculated using Fisher's exact test. The hypogonadism threshold of <8.0 nmol/L was defined according to Arver and Lehtihet. Significant differences are indicated by P-values < 0.05. Values in bold are significant P-values

**Adiposity markers and their correlation with serum testosterone concentrations:** Table 4 presents the correlations between anthropometric measures and serum total testosterone concentrations in both groups. Weight revealed a significant negative correlation with testosterone in the non-heroin-injecting men (Spearman's ρ = -0.489, p = 0.046), while the heroin injecting group showed no significant correlation (Spearman's ρ = -0.054, p = 0.525). Hip circumference was positively correlated with testosterone levels in the heroin injecting group, with a significant positive correlation (Spearman's ρ = 0.173, p = 0.039), while in the non-heroin-injecting group, the correlation did not reach statistical significance (Spearman's ρ = 0.475, p = 0.054), though the moderate positive coefficient may reflect limited statistical power given the small sample size (n = 17). Similarly, WHR showed a significant negative correlation in the heroin injecting group (Spearman's ρ = -0.204, p = 0.014), but no significant association was found in the non-heroin injecting group (Spearman's ρ = -0.212, p = 0.414).

**Table 4:** association of occupational, substance use, and anthropometric measures with serum total testosterone concentrations among heroin-injecting (n=143) and non-heroin-injecting (n=17) men in Mombasa County, Kenya, July 2012 to February 2013

Variable	Non-heroin-injecting men, n=17	P-value	Heroin-injecting men, n=143	P-value
**Occupation**				
Hospitality	0.027 (0.065)	**0.048**	0.025 (2.375)	**0.001**
Small business	0.860 (2.595)		3.690 (1.555)^a^	
Transport	3.305 (2.980)		3.575 (1.890)^a^	
**Duration of injection, years**				
<1 year	…^b^	0.301	3.21 (1.07)	0.064
≥1 year	0.093 (2.541)		3.69 (2.319)	
Weight, kg	-0.489	**0.046**	-0.054	0.525
Hip circumference, cm	0.475	0.054	0.173	**0.039**
WHR	-0.212	0.414	-0.204	**0.014**

For continuous variables, Spearman correlation coefficients (Spearman's ρ) and corresponding P-values are reported. Significant correlations are indicated by P-values < 0.05; for categorical variables, P-values were calculated using the Kruskal-Wallis test, followed by post hoc pairwise comparisons for occupation and the Mann-Whitney U test for duration of injection, with statistical significance set at P < 0.05. ^a^ P = 0.001 vs. hospitality. ^b^ cell suppressed due to small sample size (n < 5); estimates were considered unreliable and are not reported. WHR represents the waist-to-hip ratio; values in bold are significant P-values

**Occupational category, substance use duration, and testosterone concentrations: bivariate findings:** Table 4 presents the associations of occupational category and substance use duration with serum total testosterone concentrations among heroin-injecting and non-heroin-injecting men. Occupational history revealed that heroin-injecting men working in the hospitality sector had a significantly lower median testosterone level of 0.025 (IQR 2.375) compared to those in small businesses (median = 3.690, IQR 1.555) and the transport sector (median = 3.575, IQR 1.890) (p = 0.001). Similarly, non-heroin-injecting men in hospitality had a median of 0.027 (IQR 0.065), significantly lower than those in small businesses (median = 0.860, IQR 2.595) and transport (median = 3.305, IQR 2.980) (p = 0.048). In the analysis of data on injection duration, heroin-injecting men who had been injecting for less than one year had a median testosterone level of 3.21 nmol/L (IQR 1.07), compared to those injecting for one year or more, who had a median of 3.69 nmol/L (IQR 2.319). This difference was not statistically significant (p = 0.064). For non-heroin-injecting men, those injecting for one year or more had a median of 0.093 (IQR 2.541), with a non-significant difference (p = 0.301).

**Independent predictors of serum testosterone concentrations: multivariable regression analysis:** Table 5 presents the multivariable linear regression analysis of independent predictors of serum total testosterone concentrations among heroin-injecting men (n = 143). The overall model was statistically significant (F (7, 135) = 6.102, p < 0.001) and explained 20.1% of the variance in serum total testosterone concentrations (Adjusted R^2^= 0.201). After adjustment for age, body mass index, cigarette smoking, and waist-to-hip ratio, employment in the small business sector (B = 3.147, 95% CI: 1.936 to 4.358, p < 0.001) and in the transport sector (B = 3.103, 95% CI: 1.889 to 4.317, p < 0.001) were each independently associated with higher testosterone concentrations relative to the hospitality sector. Duration of heroin injection of one year or more was independently associated with higher testosterone concentrations (B = 0.802, 95% CI: 0.182 to 1.421, p = 0.012). Waist-to-hip ratio was independently and inversely associated with testosterone concentrations (B = -6.146, 95% CI: -10.489 to -1.804, p = 0.006). Cigarette smoking was independently associated with lower testosterone concentrations in the adjusted model (B = -0.656, 95% CI: -1.295 to -0.017, p = 0.044). Age and body mass index were not independently associated with testosterone concentrations after adjustment (p = 0.937 and p = 0.652, respectively). All VIF values were below 5.0, confirming the absence of problematic multicollinearity.

**Table 5:** independent predictors of serum total testosterone concentrations among heroin-injecting men (n=143) in Mombasa County, Kenya, July 2012 to February 2013: multivariable linear regression analysis

Variable	B	95% Confidence interval		P-value	VIF
		**Lower**	**Upper**		
Age (years)	-0.002	-0.043	0.039	0.937	1.114
Body mass index (kg/m^2^)	-0.030	-0.161	0.101	0.652	1.025
Cigarette smoking^a^	-0.656	-1.295	-0.017	**0.044**	1.096
**Occupational category^b^**					
Small business sector	3.147	1.936	4.358	**<0.001**	4.743
Transport sector	3.103	1.889	4.317	**<0.001**	4.717
**Duration of heroin injection ≥ 1 year^c^**	0.802	0.182	1.421	**0.012**	1.070
Waist-to-hip ratio	-6.146	-10.489	-1.804	**0.006**	1.032
Adjusted R^2^ = 0.201; F (7, 135) = 6.102; p < 0.001					

B: unstandardized regression coefficient representing the change in serum total testosterone (nmol/L) for a one-unit increase in the predictor, after adjustment for all other variables in the model. 95% CI = 95% confidence interval (lower and upper bounds). VIF: variance inflation factor; values below 5.0 indicate absence of multicollinearity. Bold values indicate statistical significance at P < 0.05. All variables were entered simultaneously using the forced entry method (Enter). The model was fitted using ordinary least squares linear regression with n = 143 heroin-injecting men. ^a^: Cigarette smoking coded as 1 = current smoker, 0 = non-smoker (reference). ^b^: Occupational category entered as two dummy variables with the hospitality sector as the reference group. ^c^: Duration of heroin injection coded as 1 = one year or more, 0 = less than one year (reference).

## Discussion

This study aimed to determine how substance use patterns and adiposity markers are associated with testosterone concentrations among heroin-injecting men in Mombasa County. The higher median testosterone concentration observed in heroin-injecting men compared to non-heroin-injecting men (3.570 versus 0.097 nmol/L, p < 0.0001) suggests that active heroin injection is associated with relatively higher testosterone concentrations within this population. However, both groups had testosterone concentrations substantially below the hypogonadism threshold of 8.0 nmol/L, with 97.9% of heroin-injecting men and all non-heroin-injecting men classified as hypogonadal. The difference observed between the two groups therefore reflects a relative difference within a population that is broadly testosterone-deficient by established clinical criteria, and should not be interpreted as indicating that testosterone concentrations in heroin-injecting men were clinically normal or sufficient. This is consistent with Janke *et al*. [51], who reported that male patients with opioid dependence exhibited significantly reduced serum testosterone levels relative to non-dependent controls. Notably, the study identified a significant relationship between testosterone levels and the severity of heroin craving, suggesting that disrupted testosterone metabolism may play a contributory role in opioid-related craving and dependence [51]. The near-universal hypogonadism documented in 97.9% of heroin-injecting men and in all non-heroin-injecting men carries substantial clinical consequences for this study population. Heroin-injecting men had median testosterone concentrations of 3.57 nmol/L.

These low levels are consistently associated with sexual dysfunction, including low libido and erectile difficulties, and fatigue and reduced physical performance, which together contribute to marked impairment in daily functioning [52]. Mood disturbance, depressive symptoms, and reduced vitality are also more prevalent in hypogonadal men and significantly diminish quality of life [53]. In our present study, these effects are likely magnified by concurrent undernutrition. Forty-five point five percent (45.5%) of the heroin-injecting men were underweight, with a median BMI of 18.69 kg/m^2^. This energy deficit, particularly when combined with physiological stress, can profoundly depress the hypothalamic-pituitary-gonadal axis in men [54]. When heroin-induced hypothalamic-pituitary suppression and undernutrition co-exist, the testosterone deficit is likely deeper and less amenable to spontaneous recovery than either condition would produce independently. Hypogonadism of this level is also associated with depressive symptoms [55], which may reinforce the continued heroin use as a coping behaviour, thereby sustaining the hormonal suppression itself. Despite this burden, hypogonadism is not currently screened for or treated within harm reduction programmes serving PWID in Kenya. The relatively higher testosterone concentrations observed among heroin-injecting men in the present study were initially considered consistent with the high prevalence of concurrent cigarette smoking in this group (70.6%), given that published evidence has associated tobacco use with elevated testosterone concentrations in men, attributed in part to cotinine-mediated inhibition of testosterone degradation [16].

However, the multivariable linear regression analysis revealed that, after adjustment for occupational category, age, BMI, waist-to-hip ratio, and duration of heroin injection, cigarette smoking was independently associated with lower testosterone concentrations (B = -0.656, 95% CI: -1.295 to -0.017, p = 0.044). This reversal indicates that the apparent positive association between smoking and testosterone at the bivariate level was driven by confounding, most likely by occupational category, rather than reflecting a true independent effect. This finding is consistent with one recent population-based study reporting lower total serum testosterone in male smokers [20]. The independent effect of smoking on testosterone in this population, therefore, warrants cautious interpretation, and future studies should examine tobacco and opioid interactions on the hypothalamic-pituitary-gonadal axis in greater detail. The majority of heroin-injecting men in this study identified as heterosexual (76.9%), which may be relevant given that sexual activity and partnership status have been associated with testosterone variation in some populations [56]. However, the present study did not assess partnership arrangements in detail, and this relationship warrants further investigation. Altogether, the testosterone concentrations observed in this population appear to reflect the combined influence of heroin use, concurrent substance use, occupational exposures, and lifestyle factors.

In the present study, heroin-injecting men engaging in transport and small business occupations had significantly higher testosterone levels, suggesting that men working in physically demanding jobs have relatively higher testosterone levels as compared to non- heroin-injecting men. These findings align with findings from studies that showed higher serum testosterone levels in men engaging in moderate-to-intense physical activity [57]. In contrast, several studies have indicated that individuals working in the small business and transport sectors experience higher levels of stress [58,59]. For example, research on small- and medium-sized enterprise owners identified significant stressors (effort-reward imbalance and work-family conflict), both of which were significantly associated with heightened psychological distress and increased presenteeism [60]. The findings of this study may be partly explained by the fact that a significant proportion of participants were employed in the transport sector (44.8%) and small business sector (49.0%). These occupations typically involve moderate to intense physical activity, which may have influenced hormonal levels, leading to relatively higher testosterone levels in men in the heroin injecting group. Notably, substance-related behaviors including both use and, in certain contexts, trade are common in the transport and small business sectors. This is consistent with previous studies, which showed that 24.5% and 19.9% of PWID were working in the transport and small business sectors, respectively [8]. Additionally, studies conducted in Addis Ababa, Ethiopia, showed a 40.9% prevalence of injection substance use among individuals working in the small trade [61]. Moreover, the transport and small business sectors serve as avenues for substance distribution. This assertion is supported by previous studies conducted in Tanzania, which indicated that PWID predominantly worked in the transport sector as touts and loaders, as well as in small businesses as fishmongers.

These individuals often congregated at bus stops, transport corridors, and low-income residential areas [62]. Likewise, studies on the transition to heroin injection in Kenya showed that 34.8% of men who were heroin merchants worked in the informal or criminal economy [63]. Overall, the relatively higher testosterone levels in heroin-injecting men working in the transport and small business sectors suggest a higher prevalence of heroin use among these individuals. The multivariable regression analysis confirmed that the associations of testosterone concentrations with occupational category, duration of heroin injection, and waist-to-hip ratio were independent of age and body mass index. These findings strengthen the bivariate observations reported in Table 4. The adjusted analysis also revealed that the apparent bivariate association between cigarette smoking and higher testosterone was reversed after adjustment, with smoking showing an independent inverse association with testosterone concentrations (B = -0.656, p = 0.044), suggesting the crude association was attributable to confounding by occupational category. The adjusted model accounted for approximately 20% of the variance in testosterone concentrations, suggesting that other unmeasured factors, including dietary intake, frequency of heroin use, other circulating hormones such as luteinizing hormone and follicle-stimulating hormone, and psychosocial stressors, may contribute to the remaining variance. Future studies should incorporate these variables to better explain the variance in testosterone concentrations in this population. The mechanism underlying the association between longer duration of heroin injection and higher testosterone concentrations in the present study is not fully established. While cotinine-mediated inhibition of testosterone degradation has been proposed as one pathway in tobacco users [16], the present regression analysis found that cigarette smoking was independently associated with lower testosterone after adjustment for occupational category and other covariates. This limits the extent to which cotinine can be invoked as the primary explanation. The observed association may instead reflect cumulative physiological adaptation, greater drug tolerance, or unmeasured lifestyle factors accompanying prolonged injection. Further mechanistic research is needed.

The findings of positive and inverse correlations of total testosterone and hip girth and WHR respectively, suggest that total testosterone levels are possible markers of central adiposity in heroin-injecting men. These findings are consistent with previous studies reporting that total testosterone levels are inversely correlated with WHR in men [30]. The regression analysis further confirmed that this inverse association between WHR and testosterone was independent of age, body mass index, cigarette smoking, occupational category, and injection duration, identifying WHR as an independent predictor of testosterone concentrations in heroin-injecting men beyond what the bivariate correlation alone could demonstrate. In contrast, studies conducted in male adults have demonstrated an inverse association, where higher hip circumference was linked to lower testosterone levels [22,30]. Altogether, the associations between serum total testosterone concentrations and both hip circumference and WHR suggest that regional fat distribution, particularly abdominal adiposity, modulates circulating testosterone in heroin-injecting men and may provide a stronger and more direct relationship with hormonal levels as revealed by previous studies [22]. Prior investigations have shown that adiponectin, a marker of adiposity, was directly associated with hip girth in ART-experienced individuals and indirectly with WHR in injection substance users [64]. Thus, the markers of central adiposity, including HC and WHR are potential biomarkers of circulating testosterone levels. Published studies have demonstrated a strong inverse relationship between visceral adiposity and testosterone levels in men [65]. Incorporating markers of central adiposity into routine assessments for heroin-injecting men may aid in detecting hormonal imbalances related to heroin use. This could inform more targeted interventions, including nutrition, exercise, and medical therapies, to address testosterone deficiencies and improve overall health in Kenya.

**Study limitations:** this study had several notable limitations. First, hormonal measurements were limited to serum total testosterone, as other key hypothalamic-pituitary-gonadal and -adrenal axis hormones, such as luteinizing hormone and follicle-stimulating hormone were not assessed. Evaluation of these hormones could offer further explanations for the homeostatic disruptions in the male reproductive hormones among PWID. Second, only information on sexual orientation was available for linking with the testosterone levels in the heroin-injecting men; furthermore, hypogonadism was defined based on testosterone level cut-offs of < 8.0 nmol/L [50], but information on the frequency of sexual intercourse, libido, and sperm analysis was not available to evaluate the testosterone-related reproductive health status. Third, dietary data were not available, which prevented an analysis of dietary factors known to influence testosterone levels. Fourth, the non-heroin-injecting reference group of 17 men was small. This was an inherent consequence of the proportionate stratification approach, which reflected the documented high prevalence of heroin injection among PWID in this region. The pooled proportion used to determine group sizes was derived by averaging two East African prevalence estimates, from Kenya and Ethiopia respectively, and this cross-national averaging is acknowledged as a methodological limitation. However, the principal findings of this study are not dependent on the size of the reference group, as the multivariable regression analysis and the central adiposity correlations were conducted within the heroin-injecting group of 143 men. Future studies should deliberately recruit larger non-heroin-injecting comparison groups, ideally through community-based sampling, to enable adequately powered between-group comparisons.

**Generalisability:** it is important to exercise caution when using these findings. Participants were recruited from a hospital-based cohort at Bomu Hospital in Mombasa using makeshift outreach methods, snowball sampling, and respondent-driven sampling. These methods may limit the representativeness of the broader population of PWID in Kenya or sub-Saharan Africa. Additionally, the predominance of participants working in the transport and small business sectors and the regional patterns of substance use observed may not reflect those in other settings. Thus, although our findings shed light on the biological and occupational factors associated with testosterone levels among PWID in coastal Kenya, additional studies are required to validate these associations in diverse populations and locations. The findings of this study point to several recommendations for future research and public health interventions. Regular screening for testosterone and other reproductive hormones should be conducted in people who inject heroin, particularly those with prolonged use, to detect potential hormonal imbalances. Health programs targeting individuals in physically demanding sectors like transport and small businesses should be implemented, focusing on stress management and substance use prevention. Additionally, incorporating measurements of central adiposity, such as WHR and HC, alongside testosterone levels, can help identify individuals at risk of metabolic and hormonal disorders. Further research is needed to explore the impact of dietary factors and other reproductive hormones on the health of people who inject heroin. Lastly, addiction treatment programs should include hormone health management, lifestyle advice, and hormone-related counselling as part of a comprehensive approach to care.

## Conclusion

This study reports that serum total testosterone concentrations were significantly higher in heroin-injecting men than in non-heroin-injecting men in Mombasa County, Kenya. However, both groups had median testosterone concentrations below the clinical threshold for hypogonadism, and this finding should be interpreted as a relative difference within a population that was broadly testosterone-deficient rather than as evidence of hormonal sufficiency. Multivariable regression analysis identified employment in the small business and transport sectors, duration of heroin injection of at least one year, and waist-to-hip ratio as independent predictors of serum total testosterone concentrations. Cigarette smoking was also independently associated with lower testosterone concentrations after adjustment, though this finding reversed the direction of the crude association and should be interpreted with caution, given the potential for residual confounding. The findings point to an urgent need to move beyond viewing heroin dependence solely as a behavioural or social problem. Addressing the biological dimension of heroin dependence alongside its behavioural and social consequences would therefore strengthen the comprehensiveness of care for this population. At the same time, the associations observed with occupation indicate that working in informal and physically demanding sectors intersects with biological risks. Effective interventions will therefore require strategies that simultaneously address endocrine health, occupational risk environments, and substance use, to ensure that clinical care for this population is both comprehensive and contextually appropriate.

### What is known about this topic

- Heroin injection suppresses testosterone through hypothalamic-pituitary-gonadal axis disruption, and low testosterone concentrations are consistently documented among men who use opioids;
- Low serum testosterone in men is independently associated with central adiposity, as measured by waist-to-hip ratio and hip circumference, and with adverse metabolic and reproductive outcomes;
- Concurrent use of substances including alcohol, cannabis, tobacco, and cocaine among men who inject heroin may compound endocrine disruption, though the direction and magnitude of these effects remain inconsistent across studies.

### What this study adds

- Serum total testosterone concentrations were significantly higher in heroin-injecting men than in non-heroin-injecting men in Mombasa County, Kenya, though both groups remained below the clinical threshold for hypogonadism, indicating a relative difference within a testosterone-deficient population;
- Employment in the small business and transport sectors and duration of heroin injection of one year or more were independently associated with higher serum testosterone concentrations among heroin-injecting men;
- Waist-to-hip ratio was an independent inverse predictor of serum testosterone, and hip circumference showed a significant positive correlation, identifying central adiposity markers as potential hormonal biomarkers in this population; cigarette smoking was independently associated with lower serum testosterone concentrations after adjustment for occupational category and other covariates, reversing the direction of the unadjusted association and indicating that the crude relationship was attributable to confounding.
